# Characterization of *Enterococcus faecalis* Phage IME-EF1 and Its Endolysin

**DOI:** 10.1371/journal.pone.0080435

**Published:** 2013-11-13

**Authors:** Wenhui Zhang, Zhiqiang Mi, Xiuyun Yin, Hang Fan, Xiaoping An, Zhiyi Zhang, Jiankui Chen, Yigang Tong

**Affiliations:** 1 State Key Laboratory of Pathogen and Biosecurity, Beijing Institute of Microbiology and Epidemiology, Beijing, China; 2 Affiliated Hospital, Academy of Military Medical Sciences, Beijing, China; University of Liverpool, United Kingdom

## Abstract

*Enterococcus faecalis* is increasingly becoming an important nosocomial infection opportunistic pathogen. *E. faecalis* can easily obtain drug resistance, making it difficult to be controlled in clinical settings. Using bacteriophage as an alternative treatment to drug-resistant bacteria has been revitalized recently, especially for fighting drug-resistant bacteria. In this research, an *E. faecalis* bacteriophage named IME-EF1 was isolated from hospital sewage. Whole genomic sequence analysis demonstrated that the isolated IME-EF1 belong to the Siphoviridae family, and has a linear double-stranded DNA genome consisting of 57,081 nucleotides. The IME-EF1 genome has a 40.04% G+C content and contains 98 putative coding sequences. In addition, IME-EF1 has an isometric head with a width of 35 nm to 60 nm and length of 75 nm to 90 nm, as well as morphology resembling a tadpole. IME-EF1 can adsorb to its host cells within 9 min, with an absorbance rate more than 99% and a latent period time of 25 min. The endolysin of IME-EF1 contains a CHAP domain in its N-terminal and has a wider bactericidal spectrum than its parental bacteriophage, including 2 strains of vancomycin-resistant *E. faecalis*. When administrated intraperitoneally, one dose of IME-EF1 or its endolysin can reduce bacterial count in the blood and protected the mice from a lethal challenge of *E. faecalis*, with a survival rate of 60% or 80%, respectively. Although bacteriophage could rescue mice from bacterial challenge, to the best of our knowledge, this study further supports the potential function of bacteriophage in dealing with *E. faecalis* infection in vivo. The results also indicated that the newly isolated bacteriophage IME-EF1 enriched the arsenal library of lytic *E. faecalis* bacteriophages and presented another choice for phage therapy in the future.

## Introduction

*Enterococcus faecalis* and *Enterococcus facium* belong to Gram-positive bacteria that commonly inhabit the lower intestinal tract, oral cavity, and vaginal tract of humans or animals. *E. faecalis* and *E. facium* are opportunistic pathogens. In a healthy individual, the two bacteria have no adverse effect on the host; however, the bacterium can cause life-threatening infection in immune-compromised patients [1–3]. Although *E. faecalis* is used as a starter for fermented food and probiotics [4], the bacterium can cause endocarditis and bacteremia, urinary tract infections, meningitis, and other infections in humans. In addition, *Enterococcus* has been found to be associated with diabetic foot infection and root canal treatment failure [5–7]. Several virulence factors have been thought to contribute to *E. faecalis* infections [3,8]. A plasmid-encoded hemolysin, called the cytolysin, is important for pathogenesis in animal models of infection, whereas cytolysin combined with high-level gentamicin resistance is associated with a five-fold increase in risk of death in human bacteremia patients [9–11]. *Enterococci* survive in very harsh environments including extreme alkaline pH (9.6) and salt concentrations. *Enterococci* resist bile salt detergents, heavy metals, ethanol, azide, and desiccation, and can grow at 10 °C to 45 °C and survive at 60 °C for 30 min [7]. Nosocomial infection caused by *E. faecalis* or *E. facium* is becoming a major concern in hospital settings because acquisition of virulence or antibiotic resistance makes this bacterium more difficult to be controlled. To date, even the so called “last defense line” of vancomycin has no inhibiting effect on some vancomycin-resistant strains of *E. faecalis* and *E. facium* [12,13]. Thus, finding an alternative agent or a strategy for treating antibiotic-resistant *E. faecalis* or *E. facium* infection has become increasingly indispensable [14]. 

Lytic bacteriophage or its endolysin has been reconsidered as a potential agent for treating clinical infection under the condition of multi-drug resistant bacteria emerging recently [15]. However, the characteristics of species-specific infection, or the narrow host spectrum of bacteriophage and inadequate methodologies in applying bacteriophage in animal assay or clinical research, greatly limit its further application in therapy or decontamination [16]. Thus, isolating new bacteriophage to enrich its therapeutic arsenal and assess its function in animal model will compensate the above shortages or limitations, and accelerate the application of bacteriophage in future therapy or decontamination. In this study, we isolated and characterized a lytic *E. faecalis* bacteriophage named IME-EF1 and its endolysin. The results indicated that bacteriophage IME-EF1 or its endolysin has potential in treating *E. faecalis* infection or contamination. 

## Results

### Phage isolation and characterization

**Figure 1:**
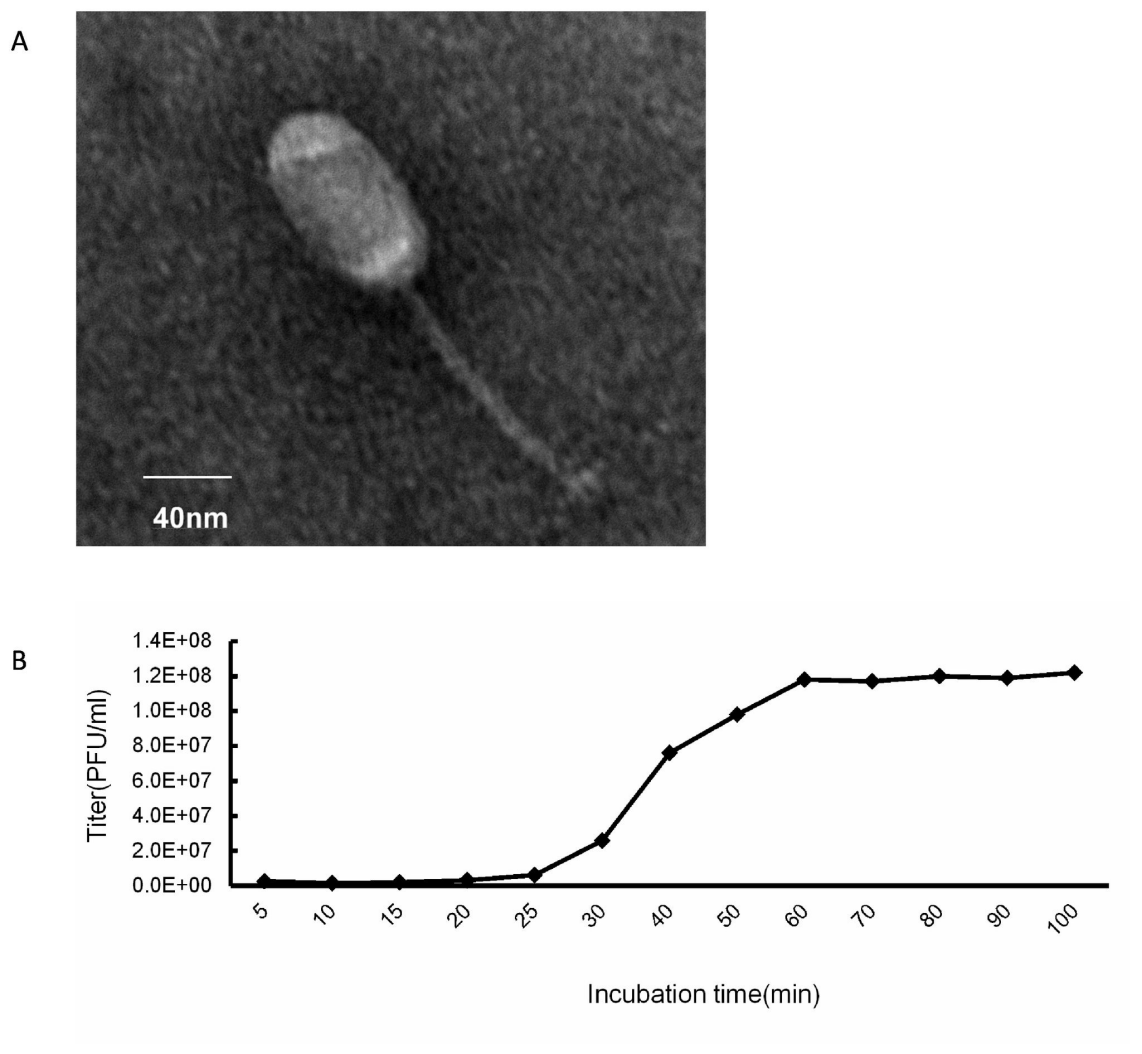
Characterization of isolated bacteriophage IME-EF1. A. Observing the morphology of bacteriophage IME-EF1 with Transmission Electronic Microscope. Magnification: ×30.0 k, bar length of 40 nm. B. One-step growth kinetics of phage IME-EF1 was determined in its host strain of *E. faecalis 002*.

When incubated with *E. faecalis 002*(Table 1), the treated sewage sample resulted in the formation of a clear plaque. Picking up the plaque and inoculating it into the *E. faecalis 002* culture at the mid-exponential phase led to the lysis of the bacteria. The isolated bacteriophage was named IME-EF1. After purification and concentration, the electron microscopy result showed that phage IME-EF1 has an icosahedral head and a non-contractile tail. The width of the head is about 35 nm to 60 nm, the length is about 75 nm to 90 nm, and the tail is about 130 nm to 220 nm (Figure 1A). Thus, the IME-EF1 was classified into the Siphoviridae family morphology. The optimal multiplicity of infection (M.O.I.) of the phage IME-EF1 was determined, as shown in Table 2. The results indicated that the same amount of phage as that of the bacteria (namely M.O.I=1) led to the highest production of progeny phage when inoculating; thus, the optimal M.O.I. of phage IME-EF1 is 1. The one-step growth kinetics is shown in Figure 1B. The results showed that the phage IME-EF1 had a latent period time of 25 min and a burst size of 60 PFU/infected bacteria when infecting its host bacteria *E. faecalis 002*. 

**Table 1:** Bacterial strains employed in this study and their antibiotic-resistant spectrum.

**Strain**	**Sample**	**Ampicillin**	**Streptomycin**	**Quinupristin**	**Nitrofurantoin**	**Linezolid**	**Moxifloxacin**	**Tigecyclin**	**Ciprofloxacin**	**Clindamycin**	**Erythromycin**	**Penicillin-G**	**Tetracyclin**	**Vancomycin**	**Levofloxacin**	**Gentamicin**
**E.faecalis 002**	Prostatitis secretions	S	S	R	S	S	S	S	R	R	R	S	R	S	S	R
**E.faecalis 019**	Prostatitis secretions	S	S	R	S	S	R	S	S	R	R	S	R	S	S	S
**E.faecalis 020**	Prostatitis secretions	R	R	R	S	S	S	S	S	R	R	S	R	S	S	R
**E.faecalis 255**	Urine	R	S	R	S	S	S	S	S	R	R	R	R	S	S	S
**E.faecalis 281**	Prostatitis secretions	S	R	S	S	S	S	S	S	R	R	S	R	S	S	R
**E.faecalis 284**	Urine	S	R	R	S	S	S	S	S	R	R	S	R	S	S	S
**E.faecalis 309**	Prostatitis secretions	S	S	R	S	S	S	S	S	R	R	S	R	S	S	S
**E.faecalis 340**	Prostatitis secretions	S	R	R	S	S	R	S	R	R	R	S	R	S	R	R
**E.faecalis V309**	Urine	S	R	R	S	S	R	S	S	R	R	S	R	R	S	R
**E.faecalis V583**	Prostatitis secretions	R	R	R	S	S	R	S	R	R	S	R	R	R	R	R
**E.faecium 010**	Sputum	R	R	S	R	S	R	S	R	R	R	R	S	S	R	R
**E.faecium 064**	Sputum	R	R	S	R	S	R	S	R	R	R	R	S	S	R	R
**E.faecium 065**	Sputum	R	R	S	R	S	R	S	R	R	R	R	S	S	R	R
**E.faecium 081**	Sputum	R	R	S	R	S	R	S	R	R	R	R	S	S	R	R
**E.faecium 106**	Sputum	R	R	S	R	S	R	S	R	R	R	R	S	S	R	R
**E.faecium 137**	Sputum	R	R	S	S	R	R	S	R	R	S	R	S	S	R	R
**E.faecium 271**	Sputum	R	R	S	R	S	R	S	R	R	R	R	R	S	R	R
**E.faecium 289**	Sputum	R	S	S	S	R	S	R	R	S	S	R	S	S	R	S
**E.faecium 322**	Urine	R	S	S	R	S	R	S	R	R	R	R	R	S	R	R
**E.faecium 327**	Urine	R	R	S	R	S	R	S	R	R	R	R	S	R	R	R
**S.aureus 037**	Sputum	R	S	S	S	S	S	S	S	S	S	R	S	S	S	S
**S.aureus 047**	Throat thrab	R	S	S	S	S	S	S	R	R	R	R	R	S	R	R
**S.aureus 069**	Sputum	R	R	S	S	S	R	S	R	R	R	R	R	S	R	R
**S.aureus 086**	Urine	R	R	S	S	S	S	S	R	R	R	R	S	S	S	R

**Table 2:** Determination of optimal multiplicity of infection.

Tube No.	Number of bacteria	Number of bacteriophage	M.O.I	Titer at 6h(PFU/ml)
1	1.0×10^6^	1.0×10^4^	0.01	5.0×10^7^
2	1.0×10^6^	1.0×10^5^	0.1	1.9×10^8^
3	1.0×10^6^	1.0×10^6^	1	8.3×10^9^
4	1.0×10^6^	1.0×10^7^	10	3.9×10^9^
5	1.0×10^6^	1.0×10^8^	100	1.4×10^9^

M.O.I Multiplicity of Infection

*E. faecalis 002* was infected with bacteriophage IME-EF1 at different M.O.I. The phage multiplication was calculated at 6 h post infection, and indicated with plaque forming unit (PFU) per milliliter culture.

### Whole genome sequencing and annotation

**Figure 2:**
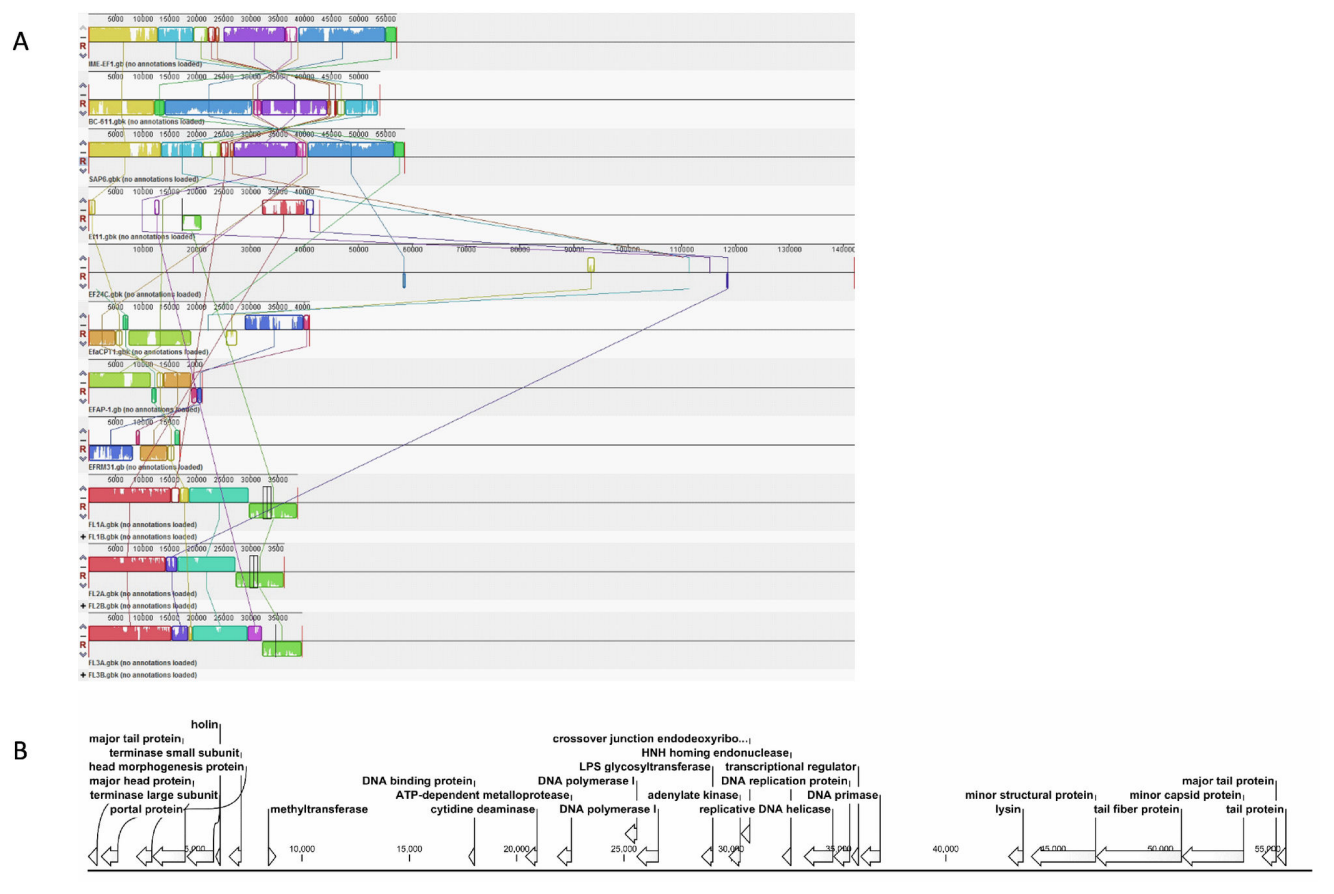
Genomic annotation and comparison of IME-EF1. A. The comparison of phage genomes from IME-EF1, EFAP-1, phiFL1-3, EFRM31, EfaCPT1, EF24c, EF11, SAP6, and BC-611 was performed using software Mauve2.3.1. B. Genomic annotation of IME-EF1 was conducted with RAST and its arrangement was illustrated using CLC Genomic Bench.

High-throughput sequencing of the phage genomic DNA generated 311,503 valid reads, with which the complete sequence of the genome was assembled using both Velvet and CLC Genomic Workbench, with an average coverage of 75× [10]. The complete genome of phage IME-EF1 consists of 57,081 bp, with an average GC content of 40.04% (GenBank Accession number: KF192053). Running BLASTN with whole genome sequence showed that the isolated IME-EF1 was highly homologous to *Enterococcus* phage SAP6 (GenBank Accession Number JF731128), with a coverage of 87%, and *Enterococcus* phage BC-611 (DDBJ Accession Number AB712291), with a coverage of 81%, both of which also belong to Siphoviridae family and were isolated in South Korea and Japan, respectively. Numerous studies on several other *E. faecalis* bacteriophage have been reported, such as EFAP-1, phiFL1-3, EFRM31, EfaCPT1 (Beheshti et al., GenBank Accession Number JX193904), EF24c, EF11, SAP6, and BC-611, which have been thoroughly genetically characterized [17–23]. However, IME-EF1 has high similarity to SAP6 and BC-611 (Figure 2A).

The IME-EF1 genome contains 98 putative CDSs, but no tRNA was found in the IME-EF1 genome using online program tRNAscan-SE 1.21 [24,25]. The culture results in the bacterium and the absence of tRNA or integrase gene in the IME-EF1 genome suggest that IME-EF1 is a lytic bacteriophage. A total of 71 CDS of IME-EF1 encode proteins of unknown function. The other 27 CDS were predicted to encode morphogenesis-related proteins (major tail protein, major capsid/head protein, phage head morphogenesis protein, phage portal protein, phage terminase large subunit, phage terminase small subunit, phage minor structure protein, tail fiber protein, minor capsid protein, and tail protein), replication-associated proteins, and DNA manipulation proteins (methyltransferase, phage DNA binding protein, cytidine deaminase, metalloprotease, DNA polymerase, LPS glycosyltransferase, adenylate kinase, endodeoxyribonuclease, HNH homing endonuclease/phage intron, replicative DNA helicase, DNA replication protein, transcription regulator, and DNA primase) (Figure 2B). 

### Endolysin identification

**Figure 3:**
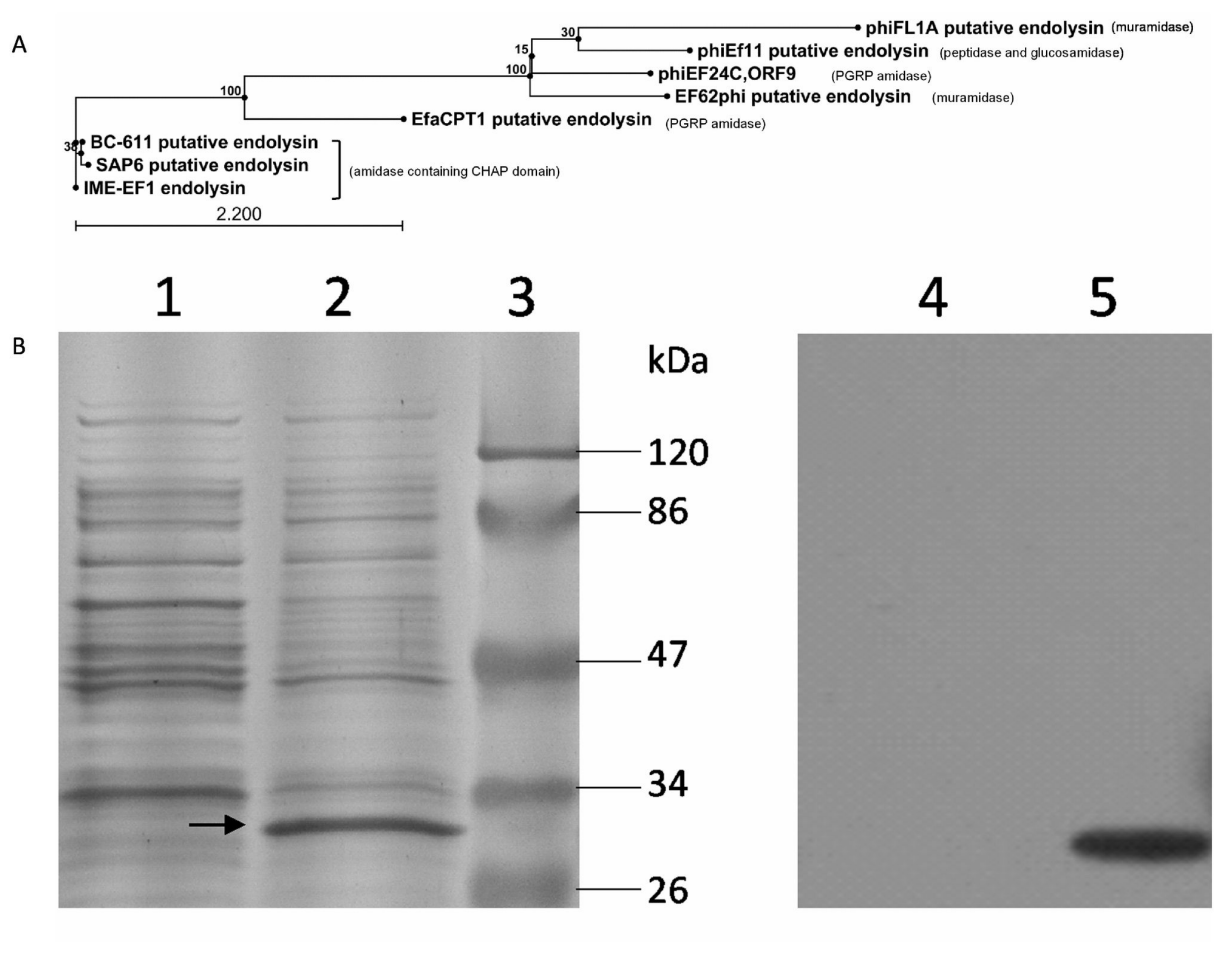
Identification of endolysin. A. Amino acid sequence of IME-EF1 endolysin was aligned and phylogenized with the submitted, confirmed, or putative endolysin from six strains of *E. faecalis* bacteriophage in GenBank. B. SDS-PAGE and Western blot were used to identify the expression of endolysin in *E. coli* M15. Lanes 1 and 4 indicate the negative control with the cells transformed with the empty vector pColdI. Lanes 2 and 5 show the cells transformed with recombinant pColdI containing IME-EF1 endolysin gene, whereas Lane 3 was pre-stained with the protein marker. Lanes 1 to 3 were stained with Coomassie brilliant blue, whereas Lanes 4 and 5 were transferred to the PVDF membrane and blotted with anti-His antibody. The black arrow indicates the expressed endolysin.

Annotation found a 714 nt length sequence in the negative strand of the IME-EF1 genome, encoding a putative 237 aa endolysin protein. The conserved domain prediction indicated that the putative endolysin is an amidase containing a conserved CHAP domain in its N-terminal end and shares highly similar amino acid sequence to the corresponding putative lysins in SAP6 and BC-611, both of which also encode a CHAP domain endolysin. When phylogenized with seven putative or confirmed endolysin from other *E. faecalis* phage, the putative endolysins from IME-EF1, SAP6, and BC-611 are located in the same sub-clade and the other containing similar domains in N-terminal sequences were also clustered (Figure 3A). The putative endolysin gene of IME-EF1 was cloned and fused with His tag sequence into vector pColdI. Through the induction of 1 mM isopropyl beta-D-thiogalactoside (IPTG), the transformed *E. coli* M15 expressed 30 kDa soluble protein, and the band can be probed using anti-His Tag antibody (Figure 3B).

### Bactericidal spectrum determination and Endolysin binding assay

**Figure 4:**
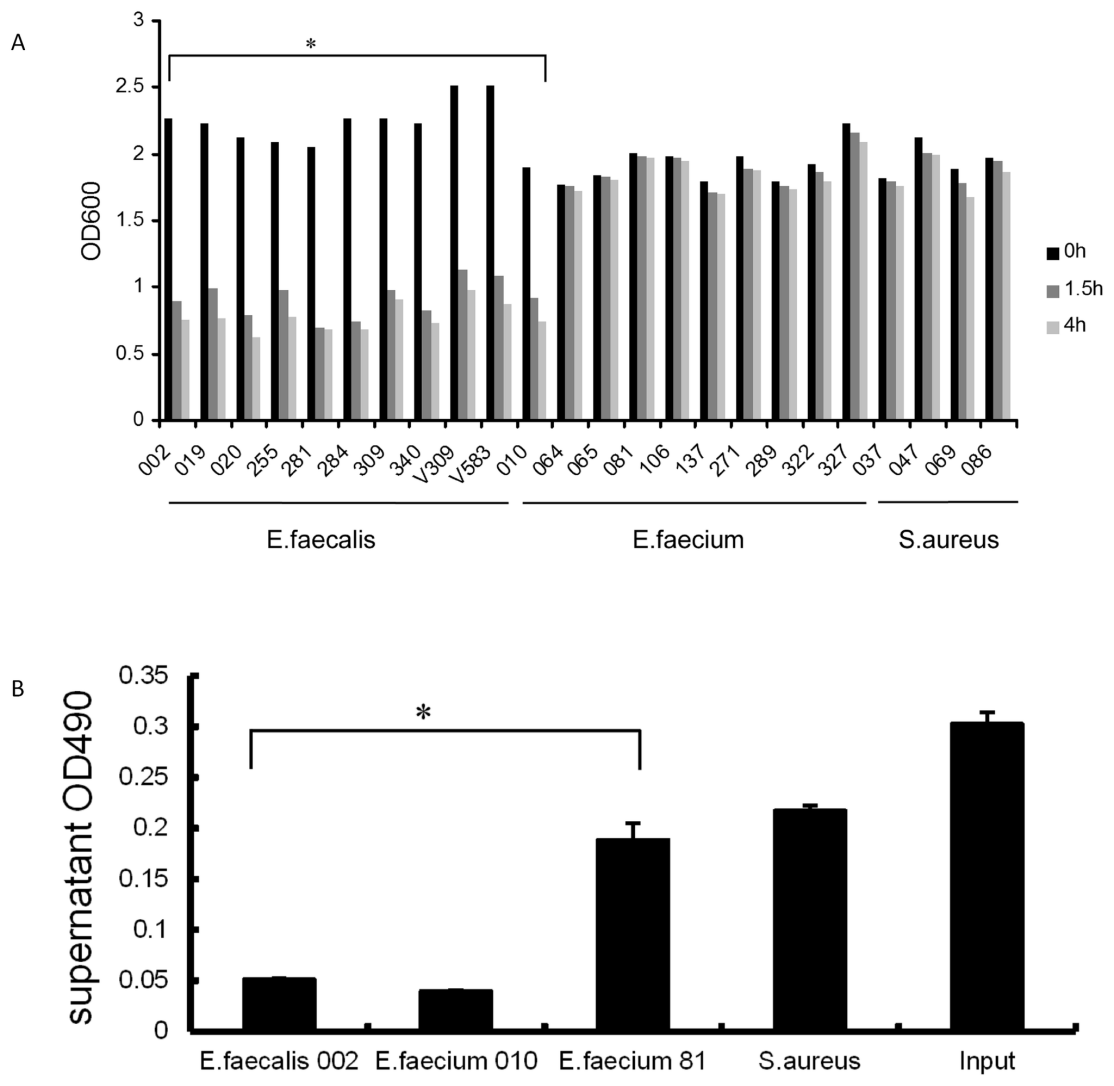
Endolysin lytic activity and binding assay. A. The OD600 descent is used to evaluate the lytic activity of IME-EF1 endolysin on the isolated *E. faecalis, E. faecium*, and *S. aureus*. B. Assay of IME-EF1 endolysin binding to sensitive bacterial strains. The expressed IME-EF1 endolysin was incubated with sensitive bacteria *E. faecalis 002* and *E. faecium 010*, as well as the insensitive bacteria *E. faecium 81* and *S. aureus* for 10 min, and the bacteria was then pelleted and the remaining supernatant endolysin was measured with ELISA at OD490. “Input” means no bacteria were added. Significance was determined using independent t test (* P<0.05).

Among the 10 clinical strains of *E. faecalis*, 10 clinical strains of *E. faecium*, and 4 clinical strains of *S. aureus*, only 3 strains of *E. faecalis* and 1 strain of *E. faecium* can be lysed by IME-EF1. However, all strains of *E. faecalis* tested in this assay and the same strain of *E. faecium* could be lysed by purified IME-EF1 endolysin (Table 3). The results further demonstrated that endolysin has wider bactericidal spectrum than its original phage. To further evaluate the bactericidal activity of IME-EF1 endolysin, bacterial culture in the mid-exponential phase was collected, suspended in PBS, and then mixed with purified endolysin. The descent of OD600 was measured and used to assess the bactericidal ability. The results showed that all the strains of *E. faecalis* used in this assay could be lysed by the purified endolysin, and resulted in the decreasing in the OD600 value mainly within 1.5 h after endolysin addition (Figure 4A).

**Table 3:** Lytic spectrum of IME-EF1 and its endolysin.

Bacterial strain	Phage	Endolysin	Bacterial strain	Phage	Endolysin
E. faecalis 002	+	+	E.faecium 065	-	-
E. faecalis 019	+	+	E.faecium 081	-	-
E. faecalis 020	+	+	E.faecium 106	-	-
E. faecalis 255	-	+	E.faecium 137	-	-
E. faecalis 281	-	+	E.faecium 271	-	-
E. faecalis 284	-	+	E.faecium 289	-	-
E. faecalis 309	-	+	E.faecium 322	-	-
E. faecalis 340	-	+	E.faecium 327	-	-
E. faecalis V309	-	+	S. aureus 037	-	-
E. faecalis V583	-	+	S. aureus 047	-	-
E.faecium 010	+	+	S. aureus 069	-	-
E.faecium 064	-	-	S. aureus 086	-	-

V Vancomycin resistant + sensitive - insensitive

The 10 strains of multi-drug resistant *E. faecalis* and *E. faecium*, as well as three strains of multi-drug resistant *S. aureus*, isolated from a hospital were used to evaluate the lytic spectrum of IME-EF1 and its endolysin on BHI agar plate. V309 and V583 were the strains of Vancomycin-resistant *E. faecalis*.

Endolysin binding assay was conducted in this study to evaluate whether binding to bacteria is required for IME-EF1 endolysin functioning during lysis. When incubated with purified endolysin, the susceptible bacterial strains can bind to endolysin. After pelleting the bacteria, the adsorbed endolysin can be pulled down accordingly, resulting in endolysin reduction in the supernatant. The bacterial strains that cannot be lysed by the endolysin, such as *E. faecium* 81 and *S. aureus*, cannot significantly reduce the supernatant endolysin compared with the sensitive bacteria or input control (Figure 4B). The trivial reduction in *E. faecium* 81 bar or *S. aureus* bar may be due to the unspecific absorption. The results showed that the lysis by IME-EF1 endolysin requires the binding of the endolysin to the host cells.

### Animal assay in vivo

**Figure 5:**
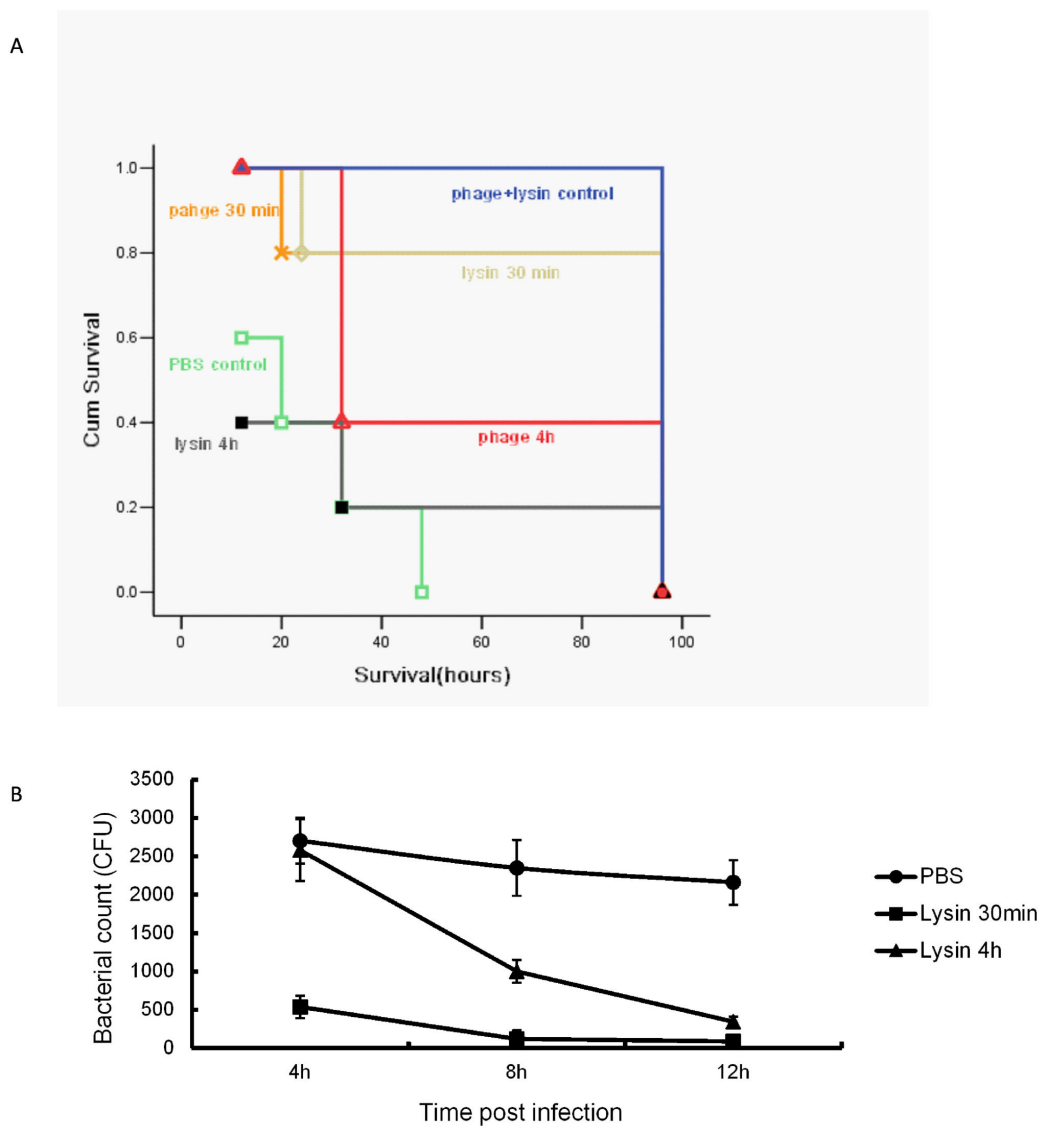
Bactericidal evaluation in mice. After intraperitoneal inoculation with *E. faecalis 002*, the phage or endolysin was administrated at 30 min or 4 h intraperitoneally, respectively. A. Mice survival rate was evaluated using Kaplan–Meier analysis. B. The tail blood samples were collected at different time points, and the bacterial number in blood was counted on BHI agar plate.

When the mice were inoculated intraperitoneally with different amount of *E. faecalis 002*, the minimum amount that resulted to death in all mice within 56 h was 2×10^9^ CFU. Thus, 2×10^9^ CFU of *E. faecalis 002* was used to construct the sepsis model in mice through intraperitoneal injection. The results indicated that one dose of bacteriophage IME-EF1 rescued the mice to some extent. Administering bacteriophage at 30 min post inoculation led to a therapeutic efficacy with a survival rate of 60%, whereas administering at 4 h provided a 40% protection rate (Figure 5A). Similar rescue pattern was observed when the mice were administrated intraperitoneally with purified endolysin. Administering endolysin at 30 min post inoculation protected 80% of the mice from lethal challenge, but the protection efficacy was poor when endolysin was administered at 4 h. The results indicated that both phage IME-EF1 and its endolysin could make an effective protection against *E. faecalis 002* challenge in mice, whereas the early administration resulted in better therapeutic efficacy than the late administration. At 4, 8, and 12 h post infection with *E. faecalis 002*, blood samples were collected from the mice tail vein, and bacterial count was figured out on BHI culture plate. Intraperitoneal administration with phage endolysin significantly reduced the number of bacteria in the blood, and early treatment at 30 min post infection had better bactericidal effect than at 4 h (Figure 5B). No bacteria were detected on the plate of the phage treatment group (data not shown). In this assay, the administration with phage IME-EF1 and endolysin without *E. faecalis 002* inoculation had no effect on mouse survival.

## Discussion

*E. faecalis* is a Gram-positive, non-motile, facultative anaerobic microbe, formerly classified as part of the Group D Streptococcus system, and commensally inhabits the gastrointestinal tracts of humans and other mammals. *E. faecalis* has been found to be responsible for some life-threatening infections in humans, especially in nosocomial environment, where the naturally high levels of antibiotic resistance found in *E. faecalis* contribute to its pathogenicity. With the emergence of antibiotic-resistant (especially vancomycin-resistant) *E. faecalis*, alternative intervention tools or strategies have been pursued for treating these drug-resistant *Enterococci* infection in future. Under these circumstances, a therapy based on phage or its lytic component acquired reconsideration. In this research, we isolated a lytic bacteriophage IME-EF1 from hospital sewage using a strain of multi-drug resistant *E. faecalis*, and explored its potential application in fighting *E. faecalis* infection. 

Although genetic characterization of numerous *E. faecalis* bacteriophage genome have been determined in previous reports, the genomic sequence of the newly isolated IME-EF1 only shared highly homologous sequence with the recently isolated *E. faecalis* phage BC-611 and SAP6. The three strains of *E. faecalis* phage belong to the Siphoviridae family and own a similar length of genomic sequence (57,081, 53996, and 58619 nt) and G+C content (40.04%, 40.45%, and 40.00%) [22,23]. The three strains of *E. faecalis* phage can be clustered in the same sub-clade based on endolysin gene (Figure 3A) or whole genome sequence (data not shown). The mean identity of the whole genome between IME-EF1 and SAP6 is 81.46%. The minor structure protein gene, tail fiber protein gene, and DNA replicative gene are more heterologous than the whole genome sequence, and share an identity between 72% and 77%. The results demonstrated that host determining genes had a vast diversity even in highly homologous phage. Although basic biological characters have been determined, the bactericidal activity of the *E. faecalis* phage of BC-611 and SAP6 had not been evaluated yet. In this study, we characterized IME-EF1 and explored its potential application in controlling *E. faecalis* infection *in vitro* and *in vivo*. Considering their homology, the results obtained from this research could predict the potential bactericidal functions of BC-611 and SAP6. The sepsis mouse model was constructed in BALB/c mice through injecting *E. faecalis* intraperitoneally. One dose administration with bacteriophage IME-EF1 or its endolysin can rescue the mice from the lethal challenges of *E. faecalis*. Under the same amount of bacteriophage or endolysin, early administration at 30 min post infection presented better protection than at 4 h post infection. Early administration with bacteriophage or endolysin has been reasonably speculated to effectively control the dissemination of bacteria. The bacterial count result in blood supported the aforementioned speculation and proved that early administration of endolysin significantly reduces the amount of *E. faecalis* in the blood. Administering bacteriophage intraperitoneally also caused the significant reduction of bacteria in the blood (data not shown). However, considering the fact that bacteriophage in the blood sample possibly adsorbed to bacteria during the preparation and disturbed the subsequent clonal growth, the bacterial count of the groups rescued with bacteriophage was not presented in this study. 

The adsorption rate, latent time, and burst size are used to evaluate the phage lytic ability. The average latent time and burst size in the tailed phage are typically 40 min to 50 min and 50 PFU/infected to 100 PFU/infected bacteria, respectively [20]. The newly isolated phage IME-EF1 can form an approximately 1 mm diameter of plaque in the sensitive strains of bacteria, and has a latent time of 25 min and a burst size of 60 PFU/infected bacteria. Thus, although IME-EF1 has short latent time in host bacteria *E. faecalis*, it should be recognized as a moderately lytic phage. A broad lytic spectrum was considered as a prerequisite character of therapeutic candidate phage. In this study, although a clinical strain of *E. faecium* can be lysed by IME-EF1, the phage showed relatively narrow lytic spectrum on *E. faecalis* (3/10). *E. faecalis* phage SAP6 has been found to lyse its host bacteria, but could not lyse the other 10 strains of *E. faecalis* [23]. The results indicated that phages in the same sub-clade having homologous genomic components and arrangement may have similar bactericidal characteristics. Phage endolysin generally had broader lytic spectrum than its original phage, which was also observed in this research. The endolysin identified from IME-EF1 can lyse 10 strains of *E. faecalis* and 1 strain of *E. faecium*, which can also be infected and lysed by the original phage. Fortunately, the two strains of vancomycin-resistant *E. faecalis* that cannot be lysed by IME-EF1 could be lysed by its endolysin. 

Bacteria have evolved numerous mechanisms to counteract phage infection [26]. Endolysin has been proven to target specific molecules in the host peptidoglycan that are essential for bacterial viability [27,28]. Thus, the occurrence of endolysin-resistant bacteria is unlikely or seldom because phage have naturally evolved with their bacterial hosts over millions of years to produce the enzymes essential for the release of progeny phage. Repeated exposure of *Streptococcus pneumoniae* and *Bacillus cereus* to low concentrations of endolysin Pal and PlyG on agar plates and in liquid culture, respectively, did not result in the emergence of resistant mutants even after numerous cycles [29,30], making endolysin a hopeful candidate in treating multi-drug resistant bacterial infection in future. 

Endolysin has been generally recognized to contain a two-domain structure [31,32]. The N-terminal catalyzing domain is responsible for cleaving peptidoglycan in the host membrane, whereas the C-terminal binding domain serves to recognize the host cell and bind to it. The catalytic domain is categorized into four different groups upon the 14 cleavage sites, which include N-acetylmuramidases, N-acetyl-β-D-glucosamidases, N-acetylmuramoyl-L-alanine amidases, and L-alanoyl-D-glutamate endopeptidases [28,33]. In this research, predicting the endolysin domain was performed with NCBI specialized BLAST tool conserved domains [34]. The CHAP (cysteine, histidine-dependent aminohydrolases) domain was found in the confirmed or putative endolysins from highly homologous *E. faecalis* bacteriophage IME-EF1, SAP6, and BC-611. This domain has been previously proven to correspond to an amidase function [35]. To date, all four kinds of N-terminal catalytic domains have been found in these submitted sequences of *E. faecalis* bacteriophage putative endolysin in GenBank. The N-acetylmuramoyl-L-alanine amidase domain is found in the N-terminal of PlyV12 [36], phiEF24C ORF9 [37], and EfaCPT1 putative endolysin (GenBank Accession Number AFO10817.1). Both EF62phi (GenBank Accession Number YP_006218727) and phiFL1A putative endolysin (GenBank Accession Number YP_003347517) gained an N-terminal GH25 (glycosylhydrolase family 25) muramidase [18,38,39]. However, in contrast to EF62phi, phiFL1A putative endolysin contained a C-terminal LysM (Lysine Motif) domain, a small globular domain with approximately 40 amino acids and widespread protein module involved in binding peptidoglycan in bacteria and chitin in eukaryotes [40]. The putative amidase encoded by Enterococcus phage phiEf11 contains both peptidase family 23 and glucosamidase domains in its N-terminal (GenBank Accession Number YP_003358818) [21]. 

No domain or motif was found to be essential in C-terminal of endolysin ΦEF24C ORF9, according to the bioinformatics analysis, but a few amino acid deletion in the C-terminal resulted in the loss of function [37]. Bioinformatics prediction did not find any conserved domain in the C-terminal of IME-EF1 endolysin, but the 50 aa deletion in this region caused the mutant protein to be insoluble in *E. coli* (data not shown). Online prediction using ProtScale on the ExPASy server revealed that the C-terminal of IME-EF1 endolysin was full of hydrophilic amino acids and the deletion may destroy its hydrophilicity and stability in the solution [41,42]. The results demonstrated that, although some conserved domains had not been identified yet in the C-terminal of IME-EF1 endolysin or ΦEF24C ORF9, its role in endolysin functioning was essential. 

## Materials and Methods

### Ethics statement

All animal studies were approved by the Institutional Animal Care and Use Committee at Beijing Institute of Microbiology and Epidemiology, Beijing, China, and performed according to institutional guidelines for animal welfare. Mice were housed (8 per cage) and maintained on a 12 h light: dark cycle (lights on at 7:00 am), with continuous access to food and water. At the end of the animal study, the mice were sacrificed by CO_2_ asphyxiation in accordance with the guideline. Bacterial isolation and sewage collection were performed in Affiliated Hospital, Academy of Military Medical Sciences by Xiuyun Yin, who is a staff of the hospital. Animal studies were performed in the Experimental Animal Center at Beijing Institute of Microbiology and Epidemiology, and did not involve any endangered or protected species. No specific permissions were required for these locations and activities.

### Phage isolation

The bacterial strains employed in this study were isolated in the Affiliated Hospital, Academy of Military Medical Sciences, Beijing, China, and shown in Table 1. Brain Heart Infusion (BHI) was purchased from Becton Dickinson and Company (Sparks, MD). The sewage water sample was collected from the same hospital, centrifuged at 10,000 × g for 10 min, and then filtered through a 0.45 µm pore membrane. Approximately 100 µl of the filtered water sample was added to 5 ml of logarithmic phase *E. faecalis 002* in the BHI culture medium, and incubated overnight at 37 °C. After centrifugation (10,000× g, 4 °C, 10 min), the supernatant was collected and filtered through a filter with 0.45 µm pore membrane. The filtrate (0.1 ml) was mixed with 0.5 ml of *E. faecalis 002* in BHI culture medium (OD600=0.6) and 5 ml of molten top soft nutrient agar (0.75% agar), which was then overlaid onto solidified base nutrient agar (1.5% agar) [43]. Following incubation for 6 h at 37 °C, the clear plaques by phage were picked from the plate. The phage titer was determined using the double-layered method previously described by Adams [44] .

### Purification and characterization of bacteriophage IME-EF1

A single plaque was picked into 5 ml of BHI medium containing *E. faecalis 002* (OD600 = 0.6) and cultured at 37 °C for 6 h. The entire 5 ml of suspension was transferred into 500 ml of BHI medium for culture at 37 °C overnight. Chloroform was then added into the 500 ml of culture to a final concentration of 0.1% before being mixing gently and allowed to stand at room temperature for about 30 min. Solid sodium chloride was added to the culture to a final concentration of 1 M, which was then incubated in an ice water bath for 1 h. The culture was centrifuged at 11,000 × g for 10 min to remove the cell debris. Subsequently, polyethylene glycol 8000 (PEG8000) was added into the supernatant to a final concentration of 10% (w/v) while slowly stirring with a magnetic stirrer at room temperature. The solution was then transferred to a polypropylene centrifuge tube in an ice water bath and incubated for at least 1 h to precipitate the phage particles. Following centrifugation (11,000 × g for 10 min at 4 °C), the phage-containing precipitate was suspended in 5 ml of SM buffer (5.8 g of NaCl, 1.2 g of MgSO4, 50 ml of 1 M Tris-HCl (pH7.5), 0.1 g of gelatin in 1000 ml of deionized water) [45]. An equal volume of chloroform was then added to separate the phage particles from PEG8000. After centrifugation at 3,000 × g for 10 min, the aqueous phase was recovered and filtered through a 0.22 µm pore-size membrane filter to remove debris. The purified phage was then stored at 4 °C. Bacteriophage was directly stained with 2% phosphotungstic acid on the carbon-coated grid and examined with a transmission electron microscope (Philips TECNAI-10).

### Determination of one-step growth curve

The one–step growth kinetics curve of IME-EF1 was measured as previously reported [46]. Briefly, a mid-exponential-phase culture of *E. faecalis 002* (OD600=0.4) was harvested and infected with bacteriophage IME-EF1 at an M.O.I. of 0.001, and then allowed to adsorb for 10 min at room temperature. The mixture was centrifuged, suspended in 5 ml of BHI medium, and cultured at 37 °C. Samples were obtained at 5 min or 10 min intervals, and immediately diluted and plated for phage titration. 

### Extraction of genomic nucleic acid of IME-EF1

Approximately 200 µl of purified phage suspension was added to the lysis solution (final concentration, 0.5% SDS, 50 µg/ml proteinase K, 20 mM EDTA), and the mixture was incubated for 1 h at 56 °C. An equal volume of saturated phenol was added to the mixture, gently vortexed, and the culture medium was centrifuged (10,000×g, 4 °C, 5 min). The upper aqueous was collected, and an equal volume of a solution (phenol: chloroform: isoamyl alcohol=25:24:1) was added to the upper aqueous. After gently vortexing, the mixture was centrifuged (10,000×g, 4 °C, 5 min). The upper aqueous was collected and added with an equal volume of isopropanol. The mixture was allowed to stand at −20 °C for more than 30 min, and then centrifuged (10,000×g, 4 °C, 20 min). The precipitation was collected and washed twice with 75% ethanol. The nucleic acids were suspended in deionized water and stored at −20 °C.

### Whole genome sequencing and bioinformatics analysis

The genomic DNA of the isolated bacteriophage IME-EF1 was subjected to high-throughput sequencing using a Life Technologies Ion Personal Genome Machine Ion Torrent sequencer (San Francisco, CA) according to the instructions of the manufacturer. The complete genome sequence of the phage was assembled using Velvet [47] and CLC Bio (Aarhus, Denmark), and then annotated using the online tool RAST [48]. Sequence similarity analysis was performed using the NCBI BLAST program (National Library of Medicine). Finding domains in endolysin protein sequence was conducted with NCBI Specialized BLAST in *CCD* (conserved domain database). The alignment of putative endolysin proteins was carried out with software CLC Bio in the parameter of less accurate, Gap open cost 10, and Gap extension cost 1.

### Identification and characterization of the phage endolysin

The genome of the phage was annotated with the online tool RAST. The coding sequence of the putative endolysin of the phage was amplified with primers (Forward: 5'-gcggatccatggttaaattaaacgatgtac-3'; Reverse: 5'-gctctagactatactttaacttgtggtaaagc-3'). The PCR fragment was cloned into the expression vector pColdI using restriction endonuclease XbaI and BamHI (NEB). The plasmid was sequenced and transformed into *E. coli* strain M15 for expression. When the transformed *E. coli* grew to an optical density 0.6 at 600 nm under the selection of 100 µg/ml ampicillin, the suspension was allowed to stand for 30 min at 16 °C. The growth medium was supplemented with IPTG to a final concentration of 1 mM, and cultured aerobically for 24 h at 16 °C. After centrifugation (10,000×g, 10 min, 4 °C), the cell pellets were collected, and then stored at −70 °C. To check the expression of the putative endolysin gene in *E. coli*, the cell pellet was sonicated and centrifuged (10,000×g, 5 min). The supernatant was dissolved in loading buffer (50 mM Tris-HCl, 2% sodium dodecyl sulfate-polyacrylamide, 0.1% bromophenol blue, 10% glycerol, and 1% β-mercaptoethanol), and then boiled for 5 min. The samples were separated on a 12% sodium dodecyl sulfate-polyacrylamide gel electrophoresis (SDS-PAGE), and stained with Coomassie brilliant blue, and then transferred to polyvinylidene difluoride membranes. The membrane was blocked overnight at 4 °C by incubating with 5% skim milk, followed by incubation for 2 h at 37 °C with the mouse anti-His monoclonal antibody (Sigma Aldrich). Bound antibody was detected with an HRP-conjugated goat anti-mouse IgG (Sigma Aldrich) combined with enhanced chemiluminescent reagents (Millipore, Bedford, MA, USA). For endolysin purification, the transformed *E. coli* cells were sonicated at 200 W to 300 W for 5 seconds with an 8 second interval. After being centrifuged (10,000×g, 20 min, 4 °C), the supernatant was added to 5 volumes of Ni-Native-0 buffer (50 mM NaH_2_PO4, 300 mM NaCl, pH 8.0). The mixture was loaded on the chromatography column and flowed through the column with a rate of 0.5 to 1 ml/min. The column was washed with five volumes of Ni-Native-500 (50 mM NaH_2_PO4, 300 mM NaCl, 500 mM imidazole, pH 8.0) buffer with a flow rate of 1 ml/min. The elution solution was then dialyzed against PBS buffer.

### Lytic spectrum determination

A spot test was adopted to determine the lytic spectrum of the bacteriophage IME-EF1 and its endolysin. Briefly, 10 strains of *E. faecalis*, 10 strains of *E. faecium*, and 4 strains of *S. aureus* were inoculated respectively on BHI semi-solid agar medium (0.8%). After solidification, each culture plate was divided into three grids. Approximately 2 µl of IME-EF1 and 2 µl of endolysin were dropped onto the center of a grid respectively, and the culture plate was then incubated at 37 °C for 12 h. The same amount of bacteriophage suspension buffer or endolysin elution buffer was spotted on a grid as the negative control. A clear and transparent plaque indicates that the host bacteria can be lysed by IME-EF1 or its endolysin.

### Lytic activity and binding assay of IME-EF1 endolysin

*E. faecalis*, *E. faecium*, and *S. aureus* were grown to mid-exponential growth phase, washed three times with PBS, and then suspended in 3 ml of PBS. Twenty-five microliter of the purified endolysin protein (1 µg/ml) was added into the bacterial suspension, and the mixture was cultured on a shaking platform at 37 °C. The value of OD600 was detected at 1.5 h and 4 h, respectively. To evaluate whether binding to host cell is necessary for endolysin functioning, *E. faecalis 002*, *E. faecium 010*, and a strain of *S. aureus* suspended in 200 µl of PBS in 1.5 ml Eppendorf tube were incubated with 25 µl of the purified endolysin protein (1 µg/ml) for 10 min, and then pelleted (10,000×g, 1 min). The endolysin in the supernatant was measured with Enzyme-linked immunosorbent assay (ELISA). Briefly, 25 µl of the supernatant in 75 µl of coating buffer (0.2 M sodium carbonate/bicarbonate, pH 9.4) was immobilized on a polystyrene microtiter plate (Thermo). The primary antibody is mouse anti-His monoclonal antibody, whereas the secondary is an HRP-conjugated goat anti mouse IgG antibody. TMB (tetramethyl benzene) was used as substrate, and optical absorbance is measured at 490 nm. 

### Mouse model infected with *E. faecalis 002* and therapeutic assay

Female (6 to 8 weeks old) BALB/c mice were purchased from the Laboratory Animal Center of the Academy of Military Medical Sciences, Beijing, China. *E. faecalis 002* was grown to OD600=1.6, and then centrifuged (10,000×g, 10 min, 4 °C). The bacterial pellet was suspended in saline and counted on the plate of the solid BHI culture medium. To determine the minimum lethal dosage, 5×10^7^, 1×10^8^, 2×10^8^, 5×10^8^, 1×10^9^, 2×10^9^, and 5×10^9^ CFU (Clony Forming Unit) bacteria were injected intraperitoneally in 100 µl of saline. Each concentration group contains 8 mice. The mouse sepsis model with *E. faecalis 002* was constructed by injecting the minimum lethal dosage of *E. faecalis 002* intraperitoneally. The 10 M.O.I of bacteriophage IME-EF1 or 0.2 mg of expressed endolysin in 100 µl of saline was administrated intraperitoneally on the other side of abdomen at 30min, 4 h after bacterial inoculation respectively. Then, 100 µl of saline was used as non-therapeutic control. Each group contained 8 mice. The 10 M.O.I. of the bacteriophage IME-EF1 and 0.2 mg of the expressed endolysin in 100 µl of saline was administrated intraperitoneally as negative control. All the survival mice were sacrificed by CO2 asphyxiation at 96 h post inoculation.

The value of the statistical analysis of OD600 value descent, and endolysin binding capacity data was performed using an independent t test. Survival data were analyzed using a Kaplan–Meier survival analysis with a log-rank method of statistics. P<0.05 were considered significant.
